# The Impact of a Digital Guideline Version on Schizophrenia Guideline Knowledge: Results from a Multicenter Cluster-Randomized Controlled Trial

**DOI:** 10.1192/j.eurpsy.2025.1543

**Published:** 2025-08-26

**Authors:** T. Halms, G. Gaigl, C. Lorenz, D. Güler, N. Khorikian-Ghazari, A. Röh, S. Leucht, A. Hasan

**Affiliations:** 1 Department of Psychiatry and Psychotherapy, Medical Faculty, University of Augsburg, Augsburg; 2 Department of Psychiatry and Psychotherapy, Technische Universität München, Munich; 3 Partner site Munich/Augsburg, German Center for Mental Health (DZPG), Munich/Augsburg, Germany

## Abstract

**Introduction:**

While clincial practice guidelines are an effective means of improving healthcare, they are not always adequately implemented. A recent study of the German S3 guideline for schizophrenia (version 2019) revealed low rates of adherence among medical professionals (Khorikian-Ghazari *et al.* Eur Arch Psychiatry Clin Neurosci 2023, 1-12). The factors impeding adherence are numerous and encompass individual, contextual, and guideline-related elements. The present study (Halms *et al.* 2024 BMC Medicine 2024, 22(1) 311) examines the efficacy of a digital guideline version in comparison to print/PDF formats with respect to guideline knowledge.

**Objectives:**

The primary aim of this study was to assess whether healthcare professionals using a digital version of the schizophrenia guideline achieved greater knowledge gains than those using traditional print or PDF formats. Secondary objectives included examining the usability of the formats, shared decision-making capabilities, and confidence in clinical decision-making.

**Methods:**

A multicenter, cluster-randomized study was conducted in psychiatric hospitals in South Bavaria, Germany. Medical and psychological staff were divided into two groups: Implementation of the guideline via the digital MAGICapp platform or the conventional print/PDF version. The study comprised a baseline assessment (T0) and a post-intervention assessment (T1) after a six-month implementation phase. The primary outcome measure was guideline knowledge, measured by knowledge questions about the contents of the German S3 guideline for schizophrenia.

**Results:**

A total of 217 subjects participated at the initial assessment (T0), while 120 subjects completed the follow-up assessment (T1). Both groups demonstrated notable gains in knowledge, yet no significant differences were observed between the two groups. At T0, 43.6% of the control group and 52.5% of the intervention group met the specified criterion. With regard to the primary outcome (≥ 30 of 46 knowledge questions and all five cardinal questions answered correctly), no significant difference was found at either T0 or T1 (T0: Chi²_(1)_ = 1.65, *p* = 0.199, T1: Chi²_(1)_ = 0.34, *p* = 0.561). Following the intervention, 58.2% of the control group and 63.5% of the intervention group met the primary outcome.

**Conclusions:**

Overall, a significant improvement in guideline knowledge was demonstrated throughout the implementation process. The digital guideline version did not demonstrate superiority in knowledge gain, but it did show potential advantages in shared decision-making. The results may have been influenced by familiarity with conventional formats and barriers to implementing digital applications. The study highlights the importance of needs-based, structured implementation strategies, particularly for younger practitioners with less professional experience.

**Disclosure of Interest:**

T. Halms: None Declared, G. Gaigl: None Declared, C. Lorenz: None Declared, D. Güler: None Declared, N. Khorikian-Ghazari: None Declared, A. Röh: None Declared, S. Leucht Consultant of: S.L. has received honoraria for advising/consulting and/or for lectures and/or for educational material from Angelini, Boehringer Ingelheim, Eisai, Ekademia, GedeonRichter, Janssen, Karuna, Kynexis, Lundbeck, Medichem, Medscape, Mitsubishi, Neurotorium, Otsuka, NovoNordisk, Recordati, Rovi, Teva., A. Hasan Grant / Research support from: This research project is part of the G-BA (Gemeinsamer Bundesausschuss) funded project SISYPHOS—Strukturierte Implementierung digitaler, systematisch aktualisierter Leitlinienempfehlungen zur optimierten Therapeutenadhärenz bei Schizophrenie, funding reference number 01VSF20024. Work on the manuscript was also supported by the German Center of Mental Health (grant number: 01EE2303C)., Consultant of: A.H. was a member of advisory boards and received paid speakership by Boehringer-Ingelheim, Lundbeck, Otsuka, Rovi, and Recordati. He received paid speakership by AbbVie and Advanz. He is editor of the AWMF German guidelines for schizophrenia.

